# Mammalian ECD Protein Is a Novel Negative Regulator of the PERK Arm of the Unfolded Protein Response

**DOI:** 10.1128/MCB.00030-17

**Published:** 2017-08-28

**Authors:** Appolinaire A. Olou, Aniruddha Sarkar, Aditya Bele, C. B. Gurumurthy, Riyaz A. Mir, Shalis A. Ammons, Sameer Mirza, Irfana Saleem, Fumihiko Urano, Hamid Band, Vimla Band

**Affiliations:** a Department of Genetics, Cell Biology and Anatomy, College of Medicine, University of Nebraska Medical Center, Omaha, Nebraska, USA; b Department of Biochemistry and Molecular Biology, College of Medicine, University of Nebraska Medical Center, Omaha, Nebraska, USA; c Department of Pathology & Microbiology, College of Medicine, University of Nebraska Medical Center, Omaha, Nebraska, USA; d Eppley Institute for Research in Cancer and Allied Diseases, University of Nebraska Medical Center, Omaha, Nebraska, USA; e Fred & Pamela Buffett Cancer Center, University of Nebraska Medical Center, Omaha, Nebraska, USA; f Division of Endocrinology, Metabolism and Lipid Research, Washington University School of Medicine, St. Louis, Missouri, USA; g Department of Pathology, Washington University School of Medicine, St. Louis, Missouri, USA

**Keywords:** ECD, ER, PERK, UPR, cell survival, GRP78

## Abstract

Mammalian Ecdysoneless (ECD) is a highly conserved ortholog of the Drosophila *Ecd* gene product whose mutations impair the synthesis of Ecdysone and produce cell-autonomous survival defects, but the mechanisms by which ECD functions are largely unknown. Here we present evidence that ECD regulates the endoplasmic reticulum (ER) stress response. ER stress induction led to a reduced ECD protein level, but this effect was not seen in PKR-like ER kinase knockout (PERK-KO) or phosphodeficient eukaryotic translation initiation factor 2α (eIF2α) mouse embryonic fibroblasts (MEFs); moreover, ECD mRNA levels were increased, suggesting impaired ECD translation as the mechanism for reduced protein levels. ECD colocalizes and coimmunoprecipitates with PERK and GRP78. ECD depletion increased the levels of both phospho-PERK (p-PERK) and p-eIF2α, and these effects were enhanced upon ER stress induction. Reciprocally, overexpression of ECD led to marked decreases in p-PERK, p-eIF2α, and ATF4 levels but robust increases in GRP78 protein levels. However, GRP78 mRNA levels were unchanged, suggesting a posttranscriptional event. Knockdown of GRP78 reversed the attenuating effect of ECD overexpression on PERK signaling. Significantly, overexpression of ECD provided a survival advantage to cells upon ER stress induction. Taken together, our data demonstrate that ECD promotes survival upon ER stress by increasing GRP78 protein levels to enhance the adaptive folding protein in the ER to attenuate PERK signaling.

## INTRODUCTION

The endoplasmic reticulum (ER) is a central subcellular organelle with essential roles in the synthesis, folding, and maturation of secreted and membrane proteins, biogenesis of cholesterol, calcium homeostasis, and regulation of survival and apoptosis pathways (1–10). Aberrations in these ER functions are sensed by well-conserved ER transmembrane sensors, namely, inositol-requiring enzyme 1 alpha (IRE1α), PKR-like ER kinase (PERK), and activating transcription factor 6 (ATF6), that activate homeostatic signaling pathways collectively referred to as the unfolded protein response (UPR) (11, 12). These ER stress sensors exhibit a dynamic and reversible interaction with the ER chaperone GRP78 (13). In unstressed cells, GRP78 is bound to luminal domains of UPR sensors, which maintains them in an inactive state (14). During ER stress, the increased load of unfolded proteins competes for GRP78 binding, leading to activation of UPR sensors (15) which, through intermediate signaling, evokes overlapping as well as pathway-specific responses to restore ER homeostasis and promote cell survival or, alternatively, eliminate stressed cells through apoptosis if homeostasis cannot be restored.

The PERK pathway has emerged as a key pathway in the cellular UPR as well as in homeostasis under unstressed conditions and in disease states (16–21). Release of GRP78 from PERK leads to its dimerization, autophosphorylation, and activation (15, 22, 23). A major PERK substrate is eukaryotic translation initiation factor 2 alpha (eIF2α), whose phosphorylation by PERK inactivates it, leading to a block in general cap-dependent protein translation and a consequent decrease in the protein load entering the ER (24); concurrently, PERK signaling selectively enhances the cap-independent translation of specific mRNAs, such as that for activating transcription factor 4 (ATF4) (24). ATF4 induces the expression of CCAAT/enhancer-binding protein-homologous protein (CHOP), which promotes apoptosis in response to stress (24–32). PERK-induced phosphorylation of eIF2α also inhibits cell cycle progression by reducing the levels of cyclins, and hence cyclin-dependent kinase 2 (CDK2) activity (33–35). Termination of PERK signaling together with dephosphorylation of eIF2α is required to reinitiate protein synthesis and resume the cell cycle. Thus, the PERK-mediated UPR leads to a coordinated program of cellular protection and mitigation of stress. However, PERK also contributes to an alternate outcome through CHOP-mediated activation of a cellular death pathway to eliminate severely damaged cells (24–32). Mechanisms to fine-tune the outcomes of UPR pathways are important to mitigate the negative consequences of ER stress. This is of particular importance under conditions where cells experience ER stress as part of their physiological responses, such as the case for antibody-secreting plasma cells or insulin-secreting pancreatic islet cells (36). We have identified ECD as a negative regulator of the PERK-mediated UPR.

The *Ecd* gene was first identified based on genetic mutations in Drosophila that led to reduced production of the developmentally regulated steroid hormone Ecdysone, which is synthesized in the ER, hence the designation Ecdysoneless for such fly mutants (37). The mammalian *Ecd* gene was cloned based on the rescue of growth defects in a Saccharomyces cerevisiae mutant with mutation of growth control regulatory gene 2 (*GCR2*), a glycolysis regulatory gene (38). Thus, the ECD protein was thought to be involved in mammalian glycolysis gene expression and was initially named hSGT1 (human suppressor of *gcr2*) (38). We identified the same gene in a screen for interacting partners of the human papillomavirus E6 oncoprotein and found it to interact with p53 and to transactivate p53-regulated genes (39, 40).

To elucidate the functional role(s) of mammalian ECD, we generated *Ecd*-null mice and demonstrated that homozygous deletion of *Ecd* was early embryonic lethal, while *ex vivo* Cre-mediated deletion of *Ecd* in *Ecd*^*fl/fl*^ mouse embryonic fibroblasts (MEFs) led to a proliferative block and a significant decrease in cell survival (41, 42). ECD was found to be essential for E2F target gene expression by facilitating the dissociation of the retinoblastoma RB protein from E2F and promoting the G_1_ to S phase of cell cycle progression (41). As a consequence, *Ecd*-null MEFs showed decreases in the levels of cyclins A, B1, E, and D1 and a reduction in CDK2 kinase activity and were arrested in the G_1_ phase of the cell cycle (41). Interestingly, E2F family proteins, such as E2F1, have been implicated in UPR-mediated cell death (43). In addition to its promotion of G_1_/S phase, ECD also promotes the G_2_/M phase of the cell cycle, and its knockdown induced not just a G_2_/M arrest but also apoptosis (44). Induction of the UPR not only induces apoptosis but also halts cell cycle progression in the G_1_ and G_2_ phases (33–35, 45), and these effects are mediated by PERK (33, 34), suggesting a potential connection between ECD and the PERK arm of the UPR.

More recently, we uncovered another mechanism by which ECD regulates cellular proliferation, involving its interaction with the RUVBL1 and PIH1D1 components of the prefoldin cochaperone R2TP (42), which is involved in the assembly or remodeling of a number of protein and protein-RNA complexes to regulate many physiological processes (46–52). Recently, it was reported that knockdown of the RUVBL1 component of the R2TP complex induced a cell cycle block and ER stress (53). Furthermore, ECD has been shown to associate with the stress response protein thioredoxin-interacting protein (TXNIP) (54), which is known to bind to the ER chaperone protein disulfide bond isomerase (PDI) to increase its enzymatic activity to relieve ER stress (55). Lastly, TXNIP was recently shown to be a novel component of the UPR and is regulated by the PERK pathway (56). Thus, multiple lines of suggestive evidence pointed to a potential role of ECD in the regulation of ER stress. In this study, we demonstrate that induction of ER stress by use of both chemical ER stress inducers (thapsigargin and tunicamycin) and a physiological ER stress inducer (glucose starvation) led to reduced ECD protein levels and that this effect was not seen in PERK kinase domain knockout (PERK-KO) or phosphodeficient eIF2α MEFs. Notably, ECD mRNA levels were increased, suggesting impaired ECD translation as a mechanism for reduced protein levels. Moreover, ECD localizes and coimmunoprecipitates with the unfolded protein response mediators PERK and GRP78. While ECD depletion increased the levels of phospho-PERK (p-PERK) and its downstream targets, p-eIF2α and ATF4, ECD overexpression markedly decreased their levels upon ER stress induction, whereas induction of the GRP78 protein robustly increased, suggesting an enhanced adaptive folding capacity of the ER. Knockdown of GRP78 reversed the attenuation of PERK signaling seen upon ECD overexpression. Significantly, ECD overexpression and depletion distinctly affected the survival outcome of cells upon ER stress.

## RESULTS

### Induction of ER stress leads to reduced ECD protein expression in a PERK-eIF2α-dependent manner.

**FIG 1:**
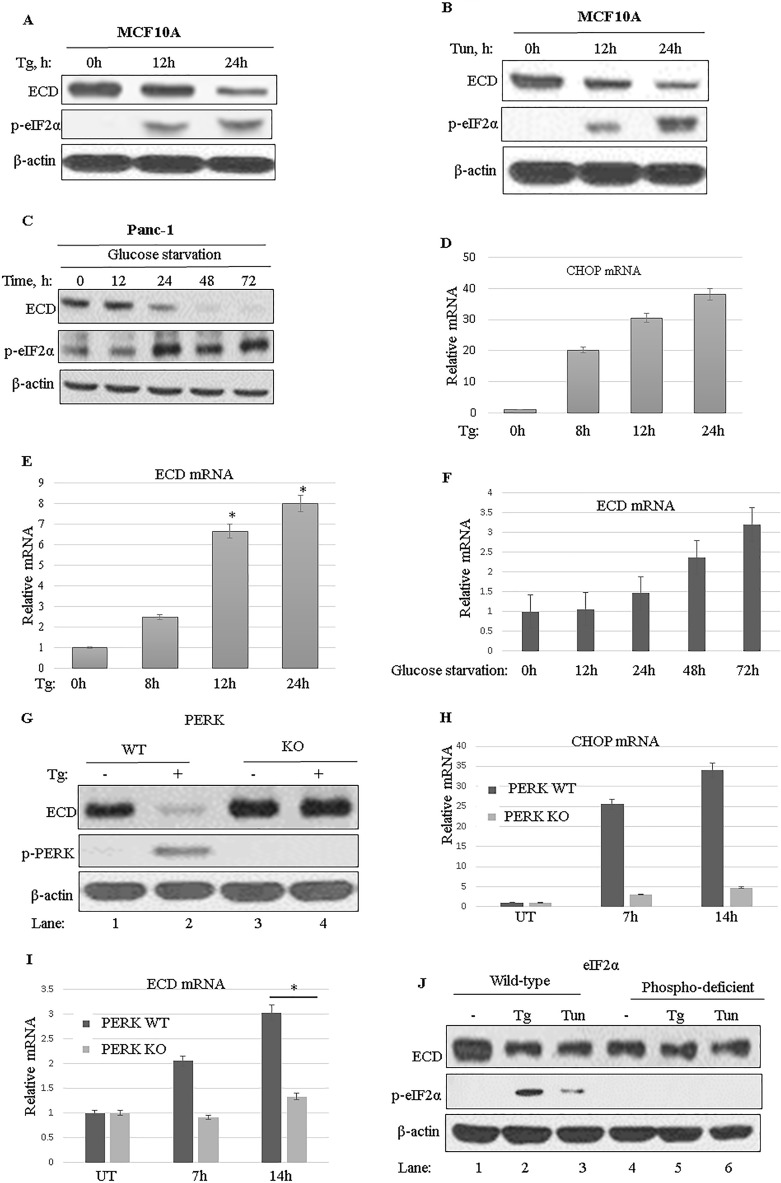
Induction of ER stress leads to reduced ECD protein expression in a PERK-eIF2α-dependent manner. (A to C) MCF-10A cells were treated with thapsigargin (Tg; 50 nM) (A) or tunicamycin (Tun; 50 ng/ml) (B), and Panc-1 cells were cultured in a glucose-free medium (C), and then cell lysates were prepared at the indicated time points. Equal amounts of proteins were resolved by SDS-PAGE and then subjected to Western blotting with the indicated antibodies. An increase in the level of p-eIF2α served as a marker for induction of ER stress. (D to F) MCF-10A cells were treated with thapsigargin (D and E) and Panc-1 cells were cultured in glucose-free medium (F), and then total RNA was isolated and subjected to qRT-PCR using CHOP primers and ECD primers. Data are means and standard deviations (SD) for 3 independent experiments *, *P* < 0.05. CHOP mRNA induction served as a control for thapsigargin-induced ER stress. (G) Wild-type (WT) PERK and PERK kinase domain knockout (PERK-KO) MEFs were treated with thapsigargin (50 nM) for 14 h, and then cell lysates were resolved in SDS-PAGE gels and subjected to Western blotting with the indicated antibodies. (H and I) PERK-KO and control WT MEFs were treated with thapsigargin, and total RNA was isolated at the indicated time points and subjected to qRT-PCR with primers targeting CHOP (H) or ECD (I). (J) WT eIF2α MEFs or mutant eIF2α phosphodeficient MEFs were treated with thapsigargin (Tg; 50 nM) or tunicamycin (Tun; 50 ng/ml) for 14 h, and then cell lysates were analyzed by Western blotting with the indicated antibodies.

To begin to explore the potential link of ECD to ER stress, we asked if the levels of ECD protein are affected by the induction of ER stress. For this purpose, the immortal human mammary epithelial cell line MCF-10A was treated with thapsigargin or tunicamycin, two commonly used chemical ER stress inducers (57), and ECD protein levels were analyzed by Western blotting. Treatment with both ER stress inducers increased the level of p-eIF2α, as expected (57) (Fig. 1A and B). Notably, a concomitant time-dependent decrease in ECD protein levels was observed upon treatment with either thapsigargin (Fig. 1A) or tunicamycin (Fig. 1B).

Given the effects of chemical ER stress inducers on ECD protein levels, we next assessed whether physiological stresses, such as glucose starvation, would have similar effects. For this purpose, we used the human pancreatic carcinoma cell line Panc-1, which is known to exhibit ER stress upon glucose starvation (57). Significantly, similar to chemical ER stress, glucose starvation-induced ER stress also led to reduced levels of ECD protein (Fig. 1C). To assess whether the decrease in ECD protein levels was due to reduced ECD mRNA levels, we measured ECD mRNA levels by using real-time quantitative PCR (qRT-PCR). Induction of CHOP mRNA was used as a control (Fig. 1D). Notably, ECD mRNA levels not only were not reduced but in fact showed an increase (Fig. 1E), suggesting that the reduction in ECD protein level was not at the transcriptional level; likewise, physiological stress by glucose starvation also increased ECD mRNA levels (Fig. 1F). Since PERK activation and subsequent phosphorylation of eIF2α mediate a translational block in response to ER stress (24), we used MEFs in which this pathway is genetically abrogated. First, we treated wild-type (WT) MEFs or MEFs from PERK-KO mice (58) with thapsigargin and analyzed ECD protein levels by Western blotting. The expected lack of PERK pathway activation in PERK-KO MEFs was confirmed by a lack of induction of PERK phosphorylation in PERK-KO MEFs compared to that in WT MEFs (Fig. 1G). Significantly, while a decrease in ECD protein levels was observed in WT MEFs treated with thapsigargin, the levels of ECD protein were unchanged in PERK-KO MEFs (Fig. 1G). ECD mRNA was then assessed in PERK-KO and control MEFs to determine whether the levels were altered upon thapsigargin treatment. Again, CHOP mRNA induction was used as a positive control (Fig. 1H). As expected, in PERK-KO MEFs, CHOP mRNA was very minimally induced upon thapsigargin treatment compared to that in control cells (Fig. 1H), since CHOP is downstream of PERK (24–32). Notably, while ECD mRNA increased in control WT MEFs, in PERK-KO MEFs, induction of ECD mRNA was low compared to that in control WT MEFs (Fig. 1I).

To further examine the role of the PERK pathway in the downregulation of ECD protein expression, we utilized MEFs from mice that carry a serine 51-to-alanine mutation in eIF2α, which prevents its phosphorylation by activated PERK and makes the cells resistant to PERK-mediated translational blocking upon ER stress induction (59). As expected, the levels of p-eIF2α increased in WT MEFs, but not in the phospho-deficient eIF2α mutant MEFs, when ER stress was induced with thapsigargin or tunicamycin (Fig. 1J, compare lanes 1 to 3 with lanes 4 to 6). Importantly, while ECD protein levels decreased in WT MEFs treated with ER stress inducers (Fig. 1J, compare lane 1 to lanes 2 and 3), the levels of ECD protein did not change in identically treated eIF2α mutant MEFs (Fig. 1J, compare lane 4 to lanes 5 and 6). Together, these results support the conclusions that ECD protein levels are downregulated upon ER stress in a PERK-eIF2α-dependent manner and that ER stress upregulates ECD mRNA levels, suggesting a regulatory link between ECD and the PERK pathway of ER stress.

### ECD colocalizes and associates with PERK and GRP78.

**FIG 2:**
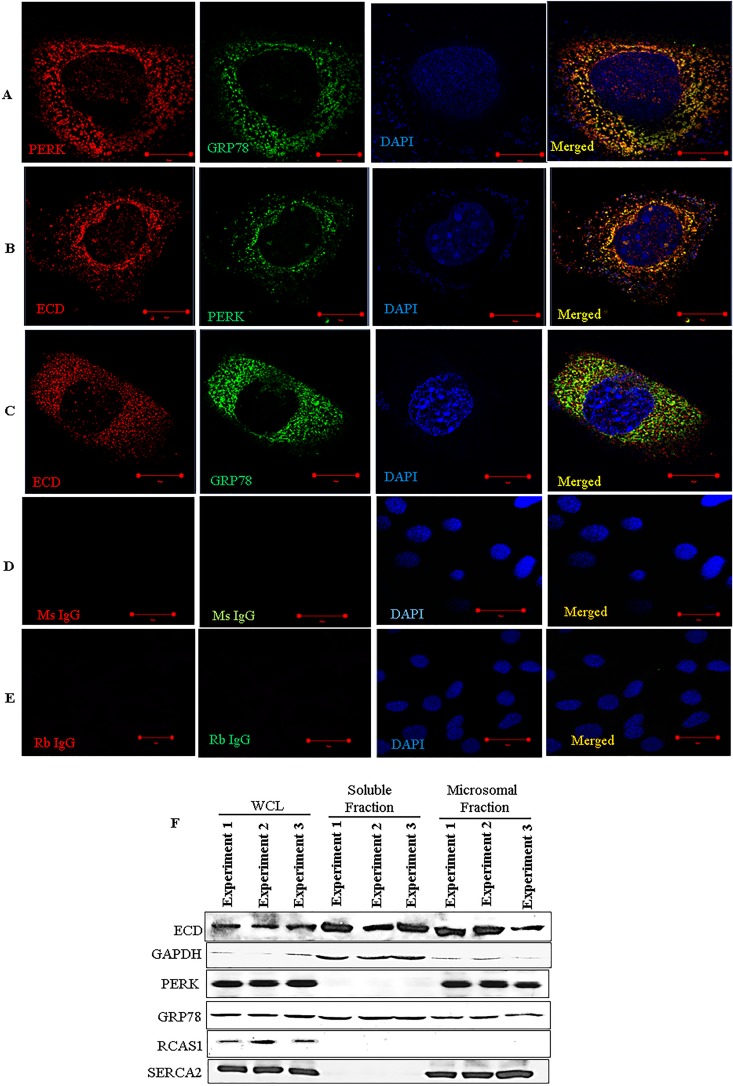

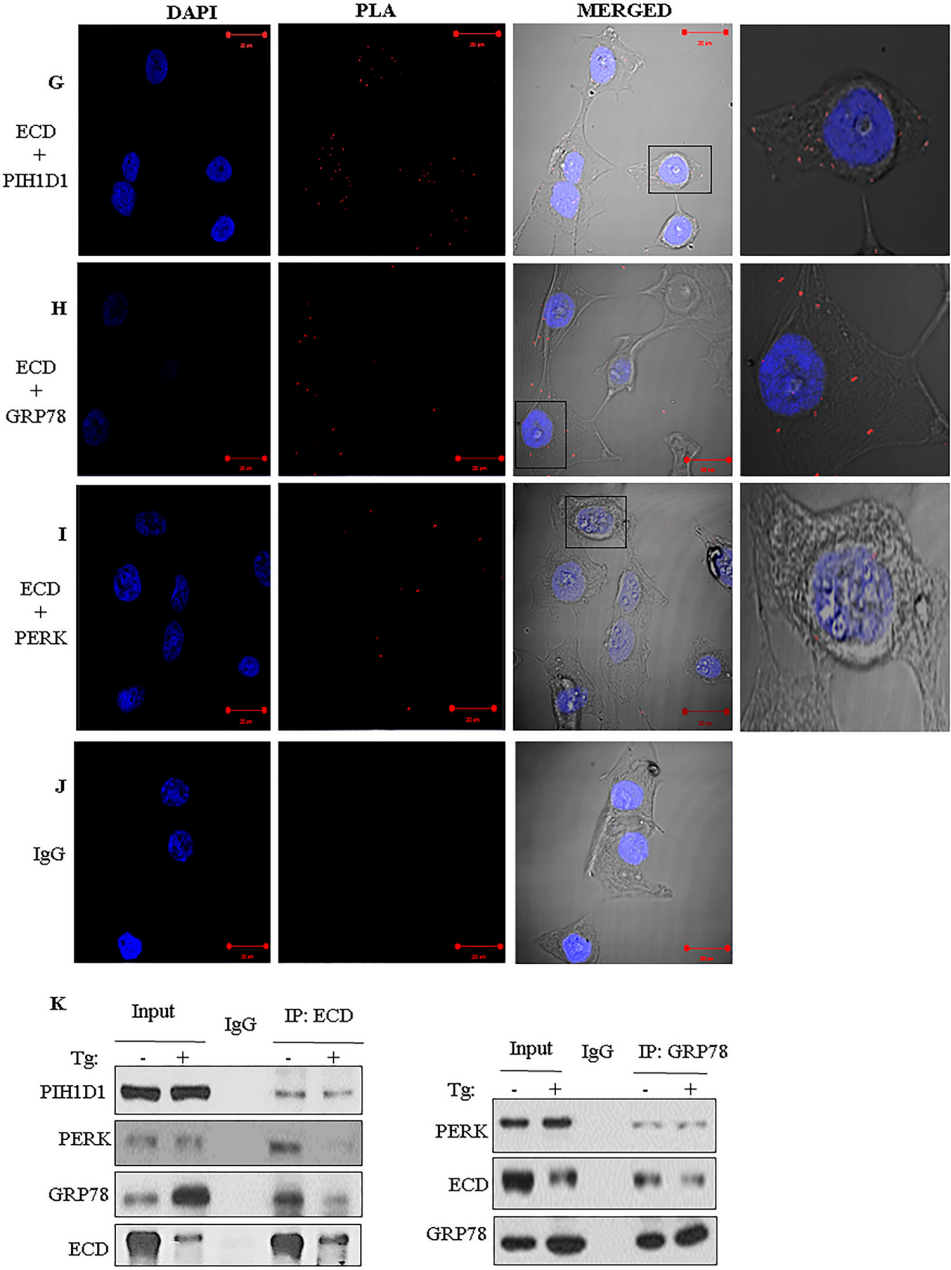
ECD colocalizes and associates with PERK and GRP78. (A to E) Immortal MEFs were fixed in 3% paraformaldehyde (PFA), stained with the indicated antibodies, and mounted for analyses by structured illumination microscopy (SIM). PERK and GRP78 colocalization served as a positive control. DAPI (4′,6-diamidino-2-phenylindole) was used to stain the nucleus. (F) MCF-10A cells were fractionated into soluble and microsomal fractions. The purity of the fractions was assessed by using GAPDH (a marker of the soluble/cytoplasmic fraction), PERK and SERCA (both markers of the microsomal fraction), and RCAS1 (a Golgi-predominant protein) as markers (88). WCL, whole-cell lysate. (G to J) MCF-10A cells were fixed with 3% PFA and stained with the indicated antibodies (all antibodies were generated in rabbits, except for the anti-ECD mouse antibody), followed by species-specific secondary antibodies linked to cDNA probes to allow fluorescent probe-based detection of the PCR amplification products as distinct foci. Incubation with proximity ligation assay plus and minus probes, followed by ligation and amplification, was carried out according to the manufacturer's protocol. Red dots indicate interactions. ECD and PIH1D1 served as positive controls. (K) Lysates of MCF-10A cells, treated with thapsigargin or left untreated, were subjected to IP with anti-ECD antibody (left) or anti-GRP78 (right), followed by Western blotting with the indicated antibodies. ECD and PIH1D1 (left) or GRP78 and PERK (right) served as positive controls.

To further explore a potential link between ECD and the ER stress pathway, we assessed the localization of ECD. We first performed immunofluorescence by using superresolution structured illumination microscopy (SIM), a technique that offers a much higher resolution than that with conventional confocal microscopy (60, 61). Colocalization of PERK and GRP78 served as a positive control, since PERK and GRP78 are known interacting partners (13). Indeed, we observed that endogenous PERK and GRP78 were colocalized in a punctate distribution (Fig. 2A). Significantly, ECD also colocalized with PERK and GRP78 (Fig. 2B and C), although not as extensively as the GRP78-PERK colocalization. Negative controls (rabbit and mouse IgGs) showed no staining (Fig. 2D and E). Next, we performed subcellular fractionation of MCF-10A cells to biochemically assess the ER localization of ECD. This analysis showed the expected presence of ECD in the soluble fraction (40, 42). Importantly, ECD was prominently present in the ER-containing microsomal fraction (Fig. 2F). Given the colocalization of ECD with PERK and GRP78 (Fig. 2A to C), we assessed if ECD associates with PERK and/or GRP78. First, we used PLA to assess the proximity of ECD to GRP78 and PERK in MCF-10A cells. A known ECD interaction with the PIH1D1 component of R2TP served as a positive control (42, 46). Indeed, ECD and PIH1D1 formed distinct foci detectable by PLA (Fig. 2G, red dots). Significantly, PLA signals were also observed for ECD and GRP78 or ECD and PERK (Fig. 2H and I). Finally, we carried out immunoprecipitation (IP) of ECD from lysates of MCF-10A cells, treated or left untreated with thapsigargin, followed by Western blotting with PERK and GRP78 antibodies to assess their association. IP of ECD coimmunoprecipitated PIH1D1 (Fig. 2K), as expected based on the known interaction of ECD with PIH1D1 (42, 46). Importantly, PERK and GRP78 also coimmunoprecipitated with ECD (Fig. 2K, left panel). A reverse IP of GRP78 also coimmunoprecipitated ECD with PERK used as a positive control (Fig. 2K, right panel). Taken together, these results suggest that ECD associates with PERK and GRP78.

### ECD regulates the PERK arm of the UPR.

The association of ECD with PERK and GRP78 (Fig. 2) and the abrogation of the decrease in ECD levels in PERK-KO cells upon ER stress (Fig. 1) suggested that ECD may be functionally linked to the UPR through the PERK pathway. Therefore, we assessed the activation of the PERK pathway in the absence or presence of UPR inducers in cells in which ECD could be inducibly deleted. For this purpose, we utilized *Ecd*^*fl/fl*^ MEFs, which were previously established and shown to exhibit *Ecd* gene deletion and loss of ECD protein expression upon infection with an adenovirus expressing Cre recombinase (adeno-Cre) (41). Previously, we observed that induction of *Ecd* deletion in these *Ecd*^*fl/fl*^ MEFs, aside from inducing cell cycle arrest, led to a decrease in cell survival even in the absence of any stress (41, 42).

**FIG 3:**
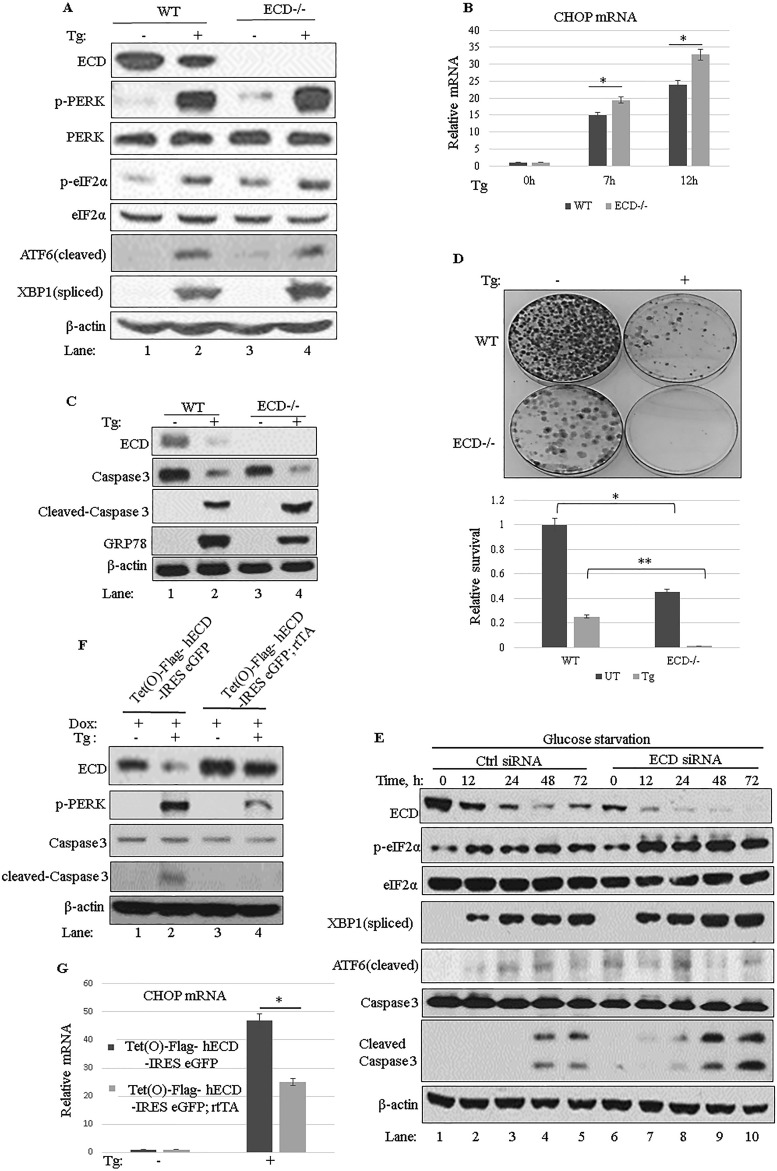

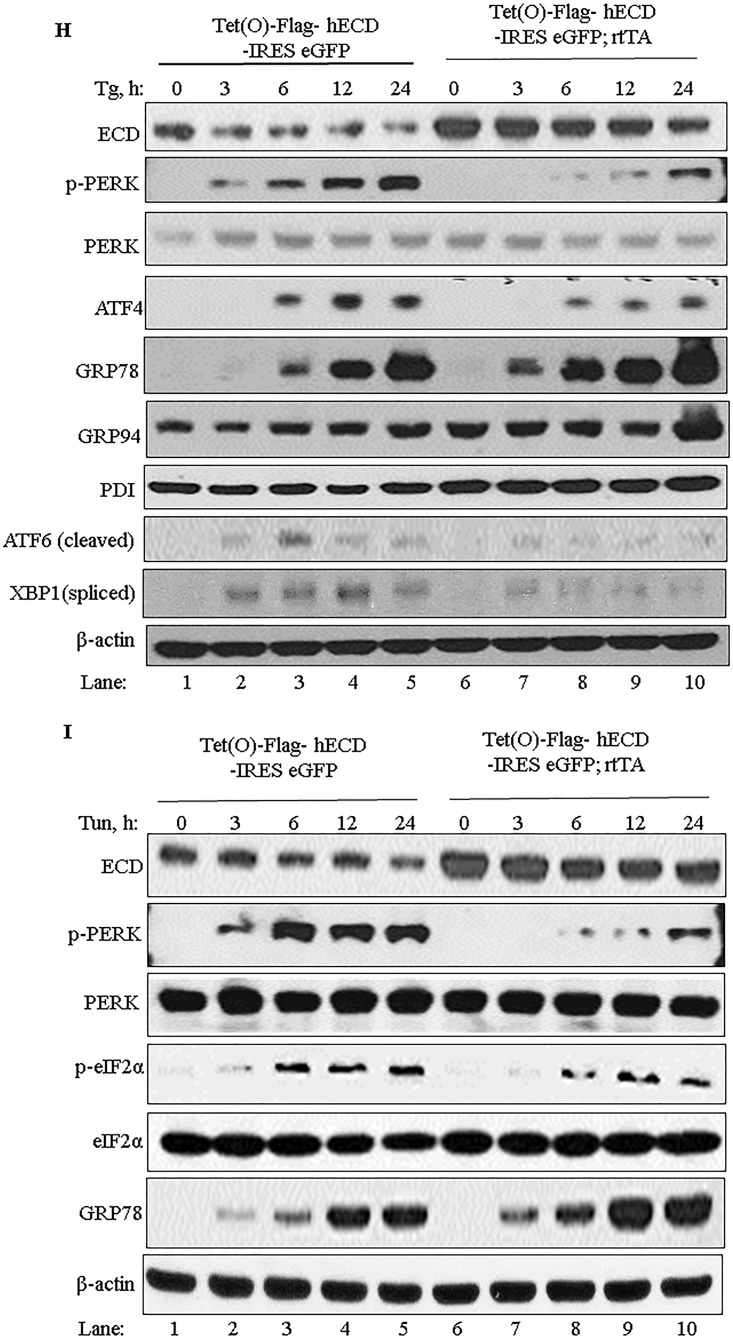
ECD regulates the PERK pathway of the UPR. (A and C) *Ecd*^*fl/fl*^ MEFs were infected with an adenovirus coding for GFP (adeno-GFP; control) or Cre (adeno-Cre) for 72 h. The cells were then left untreated or treated with thapsigargin (50 nM). Equal amounts of proteins were resolved in an SDS-PAGE gel and then subjected to Western blotting with the indicated antibodies. (B) After adenovirus infection as described for panel A, the cells were treated with thapsigargin. Total RNA was isolated and subjected to qRT-PCR with CHOP primers. The data are means and SD for 3 independent experiments. *, *P* < 0.05. (D) After adenovirus infection as described for panel A, equal numbers (1,000) of wild-type (WT) or ECD^−/−^ cells were plated in triplicate and treated with thapsigargin for 24 h. Ten days later, surviving colonies were assessed after crystal blue (0.5% in 25% methanol) staining. The color retained after the wash was dissolved in 10% acetic acid, and the absorbance at 590 nm was read. The graph in the bottom panel represents relative absorbances. The data are means and SD for 4 independent experiments. *, *P* < 0.05; **, *P* ≤ 0.002. UT, untreated cells. (E) Panc-1 cells were treated with control or ECD siRNA for 48 h. The cells were then switched to glucose-free medium, and cell lysates were prepared at the indicated time points and subjected to Western blotting with the indicated antibodies. (F, H, and I) ECD-inducible MEFs [Tet(O)-Flag- hECD-IRES-eGFP; rtTA] or control MEFs [Tet(O)-Flag-hECD-IRES-eGFP] were treated with Dox for 48 h, followed by treatment with thapsigargin (50 nM) or tunicamycin (50 ng/ml). Cell lysates were prepared at the indicated time points, and equal amounts of proteins were resolved in an SDS-PAGE gel and subjected to Western blotting with the indicated antibodies. (G) Following ECD induction and thapsigargin treatment as described above, CHOP mRNA levels were assessed using qRT-PCR.

Notably, Cre-mediated deletion of *Ecd* in the *Ecd*^*fl/fl*^ MEFs led to increases in the levels of phospho-PERK (p-PERK) and p-eIF2α compared to those in control MEFs infected with an adenovirus coding for green fluorescent protein (adeno-GFP) (Fig. 3A, compare lane 1 with lane 3), and these effects were further enhanced by treatment with thapsigargin, an inhibitor of the sarco-/endoplasmic reticulum calcium ATPase (Fig. 3A, compare lane 2 with lane 4). Spliced XBP1 also showed some increase in ECD-null cells upon thapsigargin treatment, while ATF6 did not show a significant change (Fig. 3A). Consistent with the increased p-PERK levels upon ECD deletion, thapsigargin-treated ECD-depleted MEFs also exhibited increased expression of CHOP mRNA (Fig. 3B), encoding a downstream effector of the PERK pathway whose target genes promote cell death (24–28, 30, 31). The increased CHOP mRNA level correlated with an increased level of cleaved caspase 3 in ECD-null cells (Fig. 3C, compare lane 2 with lane 4) and a significant reduction in cell survival as measured by colony formation assays (Fig. 3D). To further assess these effects, we used physiological stress in Panc-1 cells (Fig. 3E) subjected to ECD knockdown followed by glucose starvation. Again, small interfering RNA (siRNA)-mediated knockdown of ECD plus glucose starvation led to enhanced phosphorylation of eIF2α, the downstream target of PERK, and increased cleaved caspase 3 compared to that in control cells (Fig. 3E, compare lanes 1 to 5 with lanes 6 to 10), suggesting more cell death in ECD knockdown cells upon glucose starvation.

In a reciprocal approach, we overexpressed ECD and then examined its impact on the UPR, in particular the PERK pathway, upon ER stress induction. To this end, we generated MEFs from mice that carry a doxycycline (Dox)-inducible *Ecd* transgene [Tet(O)-Flag-hECD-IRES-eGFP; rtTA] or a control transgenic mouse without rtTA [Tet(O)-Flag-hECD-IRES-eGFP]. These MEFs were treated with Dox to induce ECD overexpression, followed by treatment with thapsigargin. As expected, control MEFs exhibited increases in p-PERK and CHOP mRNA; however, the MEFs with Dox-induced ECD overexpression exhibited substantially reduced levels of p-PERK and CHOP mRNA upon thapsigargin treatment compared to those in control MEFs (Fig. 3F and G). Likewise, when the MEF cell lines were treated with thapsigargin for various time points (3, 6, 12, or 24 h), ECD-overexpressing MEFs exhibited lower levels of p-PERK than those in control MEFs (Fig. 3H, compare lanes 1 to 5 with lanes 6 to 10). A corresponding reduction in the levels of ATF4, a downstream target of PERK signaling, was seen in ECD-overexpressing MEFs compared to control MEFs (Fig. 3H, compare lanes 3 to 5 with lanes 8 to 10); s-XBP1 and ATF6 levels were also slightly reduced in ECD-overexpressing MEFs (Fig. 3H, compare lanes 1 to 5 with lanes 6 to 10). Importantly, ECD overexpression was associated with substantially higher levels of GRP78 following thapsigargin treatment than those in control MEFs (Fig. 3H, compare lanes 2 to 5 with lanes 7 to 10); the levels of GRP94 protein and PDI, two other chaperones, were also slightly increased in ECD-overexpressing MEFs upon thapsigargin treatment, but not as dramatically as those of GRP78 (Fig. 3H, compare lanes 1 to 5 with lanes 6 to 10). Similar to that with thapsigargin, induction of ER stress with tunicamycin, which induces ER stress by inhibiting glycosylation of proteins in the ER, was also associated with lower levels of p-PERK and p-eIF2α in ECD-overexpressing MEFs than in control MEFs (Fig. 3I, compare lanes 2 to 5 with lanes 7 to 10). Furthermore, higher levels of GRP78 protein were also observed in ECD-overexpressing MEFs than in control MEFs upon tunicamycin treatment (Fig. 3I, compare lanes 2 to 5 with lanes 7 to 10). Taken together, these results strongly support the conclusion that ECD negatively regulates PERK signaling while enhancing the adaptive capacity of cells by increasing the levels of chaperones, predominantly the GRP78 protein, in response to ER stress.

### Increased induction of GRP78 is required for ECD to attenuate PERK signaling.

**FIG 4:**
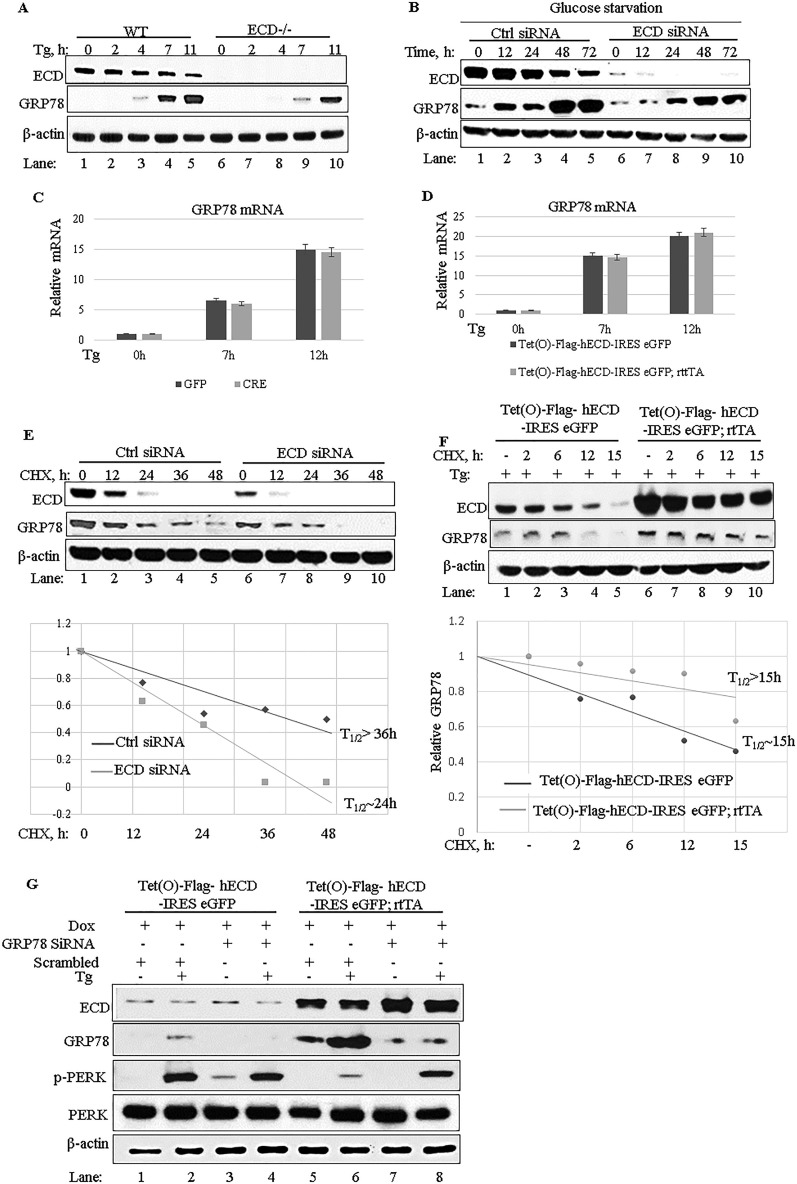
Increased induction of GRP78 expression is required for ECD to downregulate PERK signaling. (A) *Ecd*^*fl/fl*^ MEFs were treated with adenovirus as described in the legend to Fig. 3A to C, and then the cells were treated with thapsigargin (50 nM). Cell lysates were prepared at the indicated time points. Equal amounts of proteins were resolved in an SDS-PAGE gel and then subjected to Western blotting with the indicated antibodies. (B) ECD was knocked down by use of siRNA (20 nM) in Panc-1 cells, followed by exposure to glucose-free medium, and cell lysates were collected at the indicated time points and subjected to Western blotting with the indicated antibodies. (C and D) Following ECD deletion (C) or ECD overexpression (D) and thapsigargin treatment as described above, the levels of GRP78 mRNA were assessed in WT (control) versus ECD^−/−^ (adeno-Cre treated) or control versus ECD-overexpressing MEFs by use of qRT-PCR. (E) ECD was knocked down in Panc-1 cells, followed by cycloheximide treatment (25 μM). Cell lysates were prepared at the indicated time points and subjected to Western blotting with the indicated antibodies. (F) ECD-inducible MEFs and their control MEFs were treated with Dox as described previously, followed by treatment with thapsigargin and then cycloheximide treatment (25 μM) for the indicated times. Cell lysates were prepared and subjected to Western blotting with the indicated antibodies. (G) ECD-overexpressing MEFs and control MEFs were treated with GRP78 siRNA (30 nM) or control siRNA (scrambled). Twenty-four hours later, the cells were treated with Dox for 48 h to induce ECD overexpression, followed by thapsigargin treatment (50 nM). Equal amounts of proteins were resolved in an SDS-PAGE gel and subjected to Western blotting with the indicated antibodies.

Induction of GRP78 expression during ER stress is a homeostatic mechanism to reduce the unfolded protein load in the ER, thereby reducing the activation of UPR sensors. As a major regulator of PERK, overexpression of GRP78 has been shown to reduce PERK signaling upon ER stress induction (13). Given the enhanced induction of GRP78 together with attenuation of PERK signaling in ECD-overexpressing MEFs exposed to ER stress (Fig. 3H and I), we examined the possibility that the increased GRP78 levels upon ECD overexpression are linked mechanistically to the reduction of PERK signaling. To investigate this possibility, we first examined changes in the levels of GRP78 in ECD-deleted MEFs in which we observed increased PERK phosphorylation (Fig. 3A). To assess if ECD was required for optimal GRP78 protein levels upon ER stress, we exposed wild-type MEFs or MEFs with an *Ecd* deletion induced by adeno-Cre (as for Fig. 3A to D) to thapsigargin and then assessed the levels of GRP78 protein at various time points by Western blotting. The levels of GRP78 over time were reduced in ECD-null MEFs compared to the GRP78 levels in control MEFs (Fig. 4A, compare lanes 3 to 5 with lanes 8 to 10). Likewise, using physiological stress, knockdown of ECD in Panc-1 cells led to a decrease in GRP78 levels upon glucose starvation (Fig. 4B, compare lanes 1 to 5 with lanes 6 to 10). To examine whether the reduced induction of GRP78 in ECD-null MEFs was a result of reduced GRP78 mRNA expression, we carried out quantitative PCR (qPCR) analyses of mRNA isolated from WT or *Ecd*-null MEFs treated with thapsigargin. As expected, GRP78 mRNA levels increased in WT MEFs upon thapsigargin treatment (Fig. 4C); however, the levels of GRP78 mRNA induction in *Ecd*-null MEFs were comparable to those in control MEFs (Fig. 4C). Similarly, GRP78 mRNA levels in control versus ECD-overexpressing MEFs treated with thapsigargin were comparable (Fig. 4D). To determine whether GRP78 may be less stable in *Ecd*-null cells, we first knocked down ECD in Panc-1 cells, which have high levels of GRP78, followed by inhibition of protein synthesis by cycloheximide treatment for various times and analysis of GRP78 protein levels by Western blotting. Significantly, the time-dependent decrease in GRP78 protein levels following cycloheximide treatment was faster in ECD knockdown cells than in control cells (Fig. 4E). Next, to examine the possibility that ECD overexpression is associated with increased GRP78 protein stability, control or ECD-overexpressing cells were treated with thapsigargin to induce the GRP78 protein, followed by cycloheximide treatment for various times. Notably, the time-dependent decrease in GRP78 levels was slower in ECD-overexpressing MEFs than in control MEFs (Fig. 4F). Finally, to determine whether the increase in GRP78 protein levels upon ECD overexpression was required for the attenuation of PERK activation seen upon ER stress induction, we depleted GRP78 by use of siRNA in both control and ECD-overexpressing MEFs, followed by thapsigargin treatment. Notably, the attenuating effect of ECD overexpression on PERK phosphorylation upon thapsigargin treatment was abrogated by GRP78 knockdown (Fig. 4G, compare lanes 5 and 6 with lanes 7 and 8). Taken together, these results support the conclusion that ECD positively regulates the GRP78 protein level upon ER stress induction to attenuate PERK activation.

### ECD overexpression protects cells from ER stress-induced cell death.

**FIG 5:**
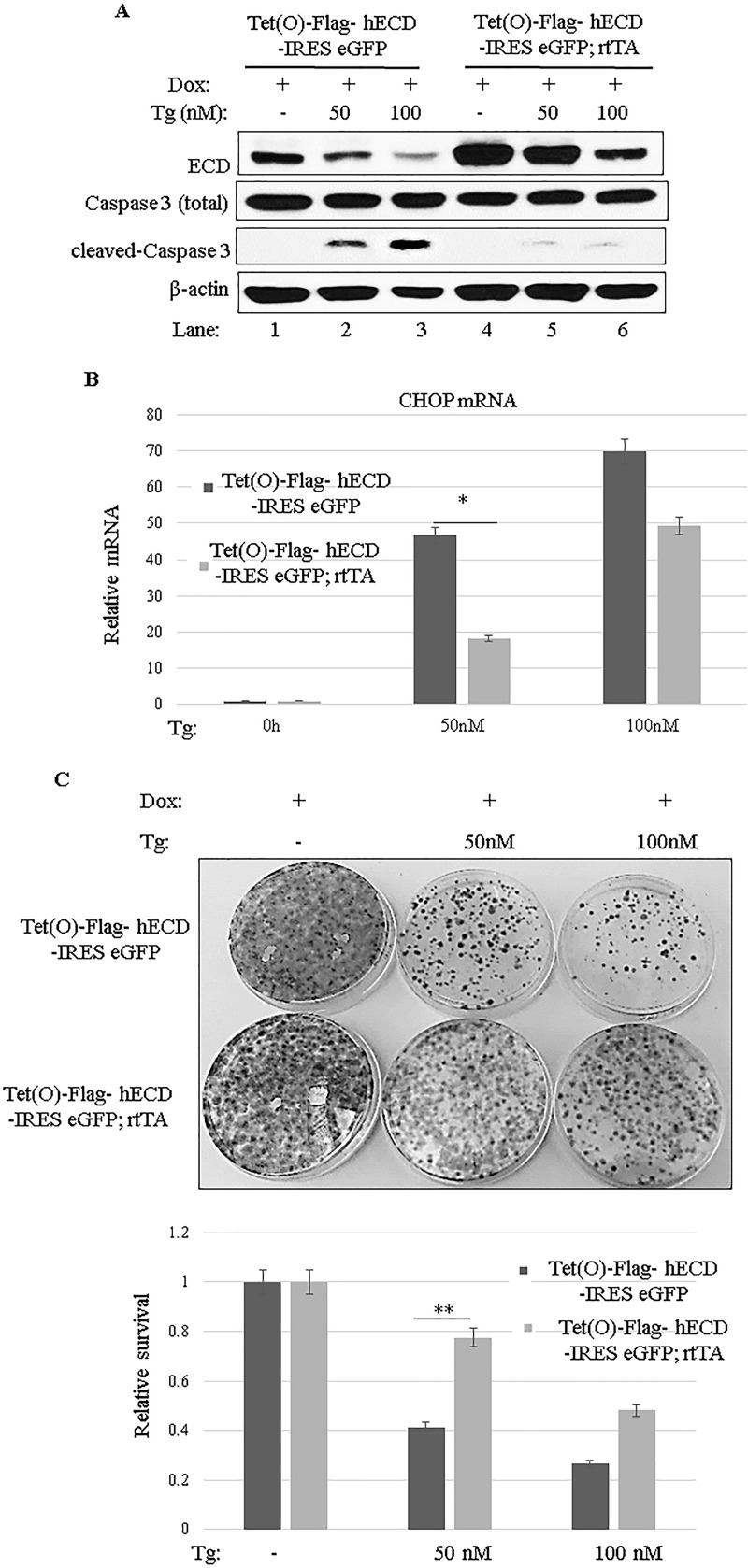
ECD overexpression provides a survival advantage. (A) ECD was induced as described in the legends to Fig. 3 and 4, followed by thapsigargin treatment for 24 h. Equal amounts of proteins were resolved in an SDS-PAGE gel and then subjected to Western blotting with the indicated antibodies. (B) Following ECD induction with Dox and thapsigargin treatment as described above, total RNA was isolated, and CHOP mRNA was assessed by qPCR. The data are means and SD for 3 independent experiments. *, *P* < 0.05. (C) After ECD induction, control and ECD-inducible MEFs were trypsinized, and equal numbers of cells (1,000) were plated in triplicate. Eight hours later, the cells were treated with thapsigargin for 24 h. Ten days later, surviving colonies were assessed by crystal blue staining (0.5% in 25% methanol). The color retained after the wash was dissolved in 10% acetic acid, and the absorbance at 590 nm was read. The graph at bottom represents relative absorbances. The data are means and SD for 4 independent experiments. **, *P* ≤ 0.002.

The ER stress response is initially aimed at the survival of cells (62); however, severe ER stress shifts the response from a prosurvival to a proapoptotic response (23, 31) through PERK-mediated induction of expression of CHOP, a transcription factor that enhances the expression of proapoptotic pathway genes (24–28, 30, 31, 63). Several studies found that inhibition/attenuation of the PERK pathway protected cells against stress-induced cell death (64–67). Given our observation that ECD functions as a modulator of PERK signaling, we assessed if the level of ECD determines a differential survival versus apoptotic cell fate upon ER stress induction. For that purpose, we treated control MEFs or Dox-inducible ECD-overexpressing MEFs with thapsigargin and then assessed the level of apoptosis induction by examining caspase 3 cleavage (68). As anticipated, a thapsigargin dose-dependent increase in cleaved caspase 3 levels was observed in control MEFs, whereas the levels of cleaved caspase 3 were markedly lower in ECD-overexpressing MEFs (Fig. 5A). Real-time qPCR analyses demonstrated that thapsigargin-induced expression of CHOP, a PERK-regulated mediator of cell death (24–28, 30–32), was lower in ECD-overexpressing MEFs than in control MEFs (Fig. 5B). To further assess the prosurvival effects of ECD against an apoptotic cell fate upon ER stress induction, we assessed the abilities of control versus ECD-overexpressing MEFs to form colonies after their exposure to ER stress. For this purpose, equal numbers of control and ECD-overexpressing MEFs were treated with thapsigargin for 24 h, and the cells were then maintained in thapsigargin-free medium for 10 days, followed by crystal violet staining and counting of surviving colonies. Notably, more colonies were observed for ECD-overexpressing MEFs than for control MEFs (Fig. 5C), further supporting the conclusion that ECD provides a survival advantage upon ER stress.

## DISCUSSION

The ER-localized stress response pathway referred to as the UPR is a well-conserved response to a number of cellular stresses, such as unfolding of proteins in the ER. The UPR elicits a spectrum of downstream responses whose outcomes range from restoration of homeostasis to cellular apoptosis if the stress is extreme and prolonged (23, 31). The UPR thus represents a double-edged sword and must be intricately regulated to prevent inappropriate cellular outcomes. Mechanisms that help to modulate the magnitude and type of UPR in response to physiological or pathological stress stimuli are not fully understood. In this study, we provide evidence that the ECD protein is a negative regulator of the PERK arm of the UPR through GRP78.

Several lines of circumstantial evidence, discussed in the introduction, suggested a potential involvement of ECD in the UPR, but direct support for such a role has been lacking. We established that induction of ER stress by use of both chemical ER stress inducers (thapsigargin and tunicamycin) and a physiological ER stressor (glucose starvation) leads to downregulation of ECD protein levels, while the mRNA levels are elevated (Fig. 1A to F). By using PERK-KO or phosphorylation-deficient eIF2α MEFs (Fig. 1G to J), we established that ECD is linked to the PERK pathway of the UPR. The association of ECD with PERK and GRP78 (Fig. 2) further linked ECD to the PERK arm of the UPR. A functional connection of ECD to the PERK arm of the UPR is supported by the distinct modulation of the PERK-mediated responses elicited by perturbations of the cellular levels of ECD. Depletion of ECD sensitized cells to PERK signaling in response to ER stress, with increases in p-PERK and p-eIF2α levels as well as an increase in the downstream effector of PERK, the transcription factor CHOP, leading to reduced survival of these ECD-depleted MEFs (Fig. 3A to E). Reciprocally, upregulation of ECD reduced PERK signaling upon induction of ER stress (Fig. 3F to I). As activation of the PERK pathway promotes cell death in response to ER stress, primarily through CHOP-dependent expression of proapoptotic genes (24–32), and inhibition/attenuation of PERK signaling protects cells against stress-induced cell death (64–66), our results support the likelihood that ECD functions to modulate PERK pathway activity and promote cell survival during ER stress. Indeed, assessment of cellular survival in response to ER stress showed that reductions in ECD levels impaired cell survival in response to ER stress (Fig. 3C to E), while overexpression of ECD promoted cell survival (Fig. 5). Collectively, these results support the conclusion that ECD and PERK are linked through a negative-feedback mechanism whereby ECD exerts an inhibitory effect on PERK pathway signaling, whereas activated PERK reduces ECD protein levels via eIF2α-dependent translational blocking. Consistent with a reciprocal negative-feedback relationship between ECD and PERK, activated PERK negatively regulates cell growth (33–35); conversely, ECD positively regulates cell growth (41) and is overexpressed in human breast and pancreatic cancer specimens, correlating with poor prognostic markers and shorter survival times (69, 70). While the physiological benefit of increased ECD mRNA upon ER stress is not yet understood, we speculate that this effect may reflect a feedback response to the decrease in ECD protein levels to compensate for the loss of ECD, but ECD translation is blocked. Although ECD has been reported to play roles in pre-mRNA splicing in Drosophila (71), it is unlikely that ECD alters its own mRNA splicing upon ER stress, because ECD protein levels decrease upon ER stress.

Modulation of ECD levels by knockdown or overexpression demonstrated that ECD is a positive regulator of GRP78 levels (Fig. 3H and I and 4). While slight increases in GRP94 and PDI levels were observed, the effect on GRP78 was more dramatic. As increased expression of GRP78 and other chaperones is known to promote clearing of the ER stress-causing unfolded protein load and to reduce the activation of UPR sensors (13–15, 55), we surmise that upregulation of GRP78 and other chaperones (Fig. 3H) may represent one potential mechanism by which ECD negatively regulates PERK pathway activation and relieves ER stress. Indeed, knockdown of GRP78 eliminated the ability of overexpressed ECD to attenuate PERK pathway signaling (Fig. 4G). Notably, GRP78 is required for cell survival not only in response to ER stress (72–75) but also under other stressful and hostile conditions, such as glucose deficiency encountered in the tumor microenvironments (76–81). Given that the PERK arm of the UPR is also activated in cancer (63, 82, 83) and that both ECD and GRP78 are overexpressed in cancer (69, 70, 76–81), we suggest that ECD overexpression may play a similar role to mitigate the negative consequences of elevated PERK signaling found in cancer. Given the mechanistic link between ECD and GRP78 in inhibiting the PERK pathway to promote cell survival, it would be of great interest to explore if GRP78 and ECD are cooverexpressed in tumors that use the UPR to promote tumor cell survival and hence may be suitable targets for UPR-directed therapeutic agents.

## MATERIALS AND METHODS

### Reagents and antibodies.

Thapsigargin and tunicamycin were obtained from Cell Signaling, dissolved in dimethyl sulfoxide (DMSO), and used at the indicated concentrations. siRNA against GRP78 was from Santa Cruz, and cycloheximide was purchased from Sigma. A monoclonal antibody against ECD, generated in our laboratory, has been described previously (69). Antibodies against p-PERK, ATF4, p-eIF2α, eIF2α, GRP78, caspase 3, GRP94, s-XBP1, RCAS1, PDI, and total PERK were from Cell Signaling. ATF6 and SERCA2 antibodies were purchased from Enzo Life and Abcam, respectively. PERK and GRP78 used for immunofluorescence and proximity ligation assay (PLA) were obtained from Santa Cruz and Abcam, respectively. PIH1D1 was from Santa Cruz (18Y9). GRP78 for IP and Western blotting was purchased from Abcam.

### Establishment of MEFs with doxycycline-inducible ECD overexpression from *Ecd*-transgenic mice.

MEFs were generated from 13.5-day-old embryos of mice of the Tet(O)-Flag-hEcd-IRES-eGFP genotype, used as a control. rtTA was introduced retrovirally to generate Tet(O)-Flag-hEcd-IRES-eGFP; rtTA mice (the derivation of the transgenic mice will be described in a separate report). The mice were handled in accordance with the Institutional Animal Care and Use Committee (IACUC).

### Cell lines and culture conditions.

*Ecd*^*fl/fl*^ MEFs were maintained in Dulbecco's modified Eagle's medium (DMEM) supplemented with 10% fetal bovine serum and treated with an adenovirus coding for GFP (adeno-GFP; control) or Cre (adeno-Cre), as described previously (41, 42). Panc-1 cells have been described previously (84). For ECD overexpression studies, control MEFs or ECD-overexpressing MEFs were cultured with 1 μg/ml of doxycycline (BD Biosciences) to induce expression of the *Ecd* transgene. MEFs with nonphosphorylatable eIF2α were obtained from the lab of Thomas Rutkowski, Carver College of Medicine, IA, and have been described elsewhere (59). PERK knockout (PERK-KO) MEFs were from ATCC (CRL-2976). The immortal human mammary epithelial cell line MCF-10A was cultured in DFCI-1 medium (42).

### Cellular fractionation and immunoprecipitation.

Subcellular fractionation of MCF-10A cells into soluble (glyceraldehyde-3-phosphate dehydrogenase [GAPDH] was used as a marker [42, 85]) and microsomal (PERK and SERCA2 were used as markers) fractions was carried out according to the manufacturer's protocol (Sigma-Aldrich) (86). For immunoprecipitation (IP), cells were washed in phosphate-buffered saline (PBS) and lysed in CHAPS buffer {0.3% 3-[(3-cholamidopropyl)-dimethylammonio]-1-propanesulfonate (CHAPS), 20 mM Tris-HCl (pH 7.4), 120 mM NaCl, 10% glycerol, 5 mM EDTA supplemented with protease and phosphatase inhibitors (Roche)}.

### Western blotting.

For Western blotting, cell lysates were prepared in radioimmunoprecipitation assay (RIPA) buffer (Thermo Scientific) supplemented with protease inhibitors (Roche). Lysates were resolved in an SDS-PAGE gel, transferred onto a nylon membrane (IPVH00010; Millipore), and subjected to enhanced chemiluminescence (ECL)-based Western blotting as described previously (41, 42).

### Immunofluorescence analysis.

Immortal MEFs were cultured on coverslips, fixed for 30 min in 3% paraformaldehyde (PFA), and permeabilized in PBS (containing 0.5% Triton X-100 and 10% goat serum) for 20 min at room temperature, followed by blocking in PBS (containing 10% goat serum) for 1 h at room temperature. The cells were then incubated with primary antibodies in blocking buffer overnight at 4°C, followed by a 1-h incubation with the corresponding secondary antibodies in blocking buffer at room temperature. Cells were then washed in PBS (containing 0.1% Tween 20) and mounted for confocal microscopy and superresolution structured illumination microscopy (SIM). Confocal images were obtained using a Carl Zeiss LSM 510 microscope, and three-dimensional (3D) SIM images were collected with an ELYRA PS.1 illumination system (Carl Zeiss).

### PLA.

PLA was performed as previously described (87). Briefly, MCF-10A cells were fixed with 3% PFA, stained with the indicated antibodies, and incubated with proximity ligation assay plus and minus probes, followed by ligation and amplification reactions according to the manufacturer's protocol (DuoLink; Sigma).

### Colony formation assay.

For clonogenic assay, 1,000 cells were plated in triplicate and allowed to attach to the plate (about 8 h), followed by treatment with thapsigargin for 24 h. Ten days later, colony formation was assessed as previously described (41, 42).

### RNA isolation and qRT-PCR.

Total RNA was isolated using Trizol reagent (Invitrogen). One microgram of RNA was reverse transcribed by use of SuperScript II reverse transcriptase (Invitrogen). qPCR was performed with the primer sets indicated in Table 1.

**TABLE 1:** Primers used for real-time quantitative PCR

Target gene	Primer sequence	
	Forward	Reverse
Mouse *CHOP*	CTGCCTTTCACCTTGGAGAC	CGTTTCCTGGGGATGAGATA
Human *CHOP*	CATTGCCTTTCTCCTTCGGG	CCAGAGAAGCAGGGTCAAGA
Mouse *GRP78*	AGTGGTGGCCACTAATGGAG	CAATCCTTGCTTGATGCTGA
Mouse *Ecd*	CCGGTCTGGCACAAACTTCTGCTG	AGGGTCGAAGCATCCCTCCATCGA
Human *Ecd*	ACTTTGAAACACACGAACCTGGCG	TGATGCAGGTGTGTGCTAGTTCCT

### Induction of ER stress by glucose starvation.

Panc-1 cells were maintained in DMEM supplemented with 10% fetal bovine serum and then switched to glucose-free DMEM (Life Technologies) for the indicated times.

### Statistical analysis.

For assessment of statistical significance, the Student *t* test was used. *P* values of <0.05 were considered statistically significant.

## References

[1] Voeltz GK, Rolls MM, Rapoport TA 2002 Structural organization of the endoplasmic reticulum. EMBO Rep 3:944–950. doi:10.1093/embo-reports/kvf202. 12370207 PMC1307613

[2] Reid DW, Nicchitta CV 2015 Diversity and selectivity in mRNA translation on the endoplasmic reticulum. Nat Rev Mol Cell Biol 16:221–231. doi:10.1038/nrm3958. 25735911 PMC4494666

[3] Jan CH, Williams CC, Weissman JS 2014 Principles of ER cotranslational translocation revealed by proximity-specific ribosome profiling. Science 346:1257521. doi:10.1126/science.1257521. 25378630 PMC4285348

[4] Ron D, Walter P 2007 Signal integration in the endoplasmic reticulum unfolded protein response. Nat Rev Mol Cell Biol 8:519–529. doi:10.1038/nrm2199. 17565364

[5] Ikonen E 2008 Cellular cholesterol trafficking and compartmentalization. Nat Rev Mol Cell Biol 9:125–138. doi:10.1038/nrm2336. 18216769

[6] Ron D, Hampton RY 2004 Membrane biogenesis and the unfolded protein response. J Cell Biol 167:23–25. doi:10.1083/jcb.200408117. 15479733 PMC2172515

[7] Fagone P, Jackowski S 2009 Membrane phospholipid synthesis and endoplasmic reticulum function. J Lipid Res 50(Suppl):S311–S316. doi:10.1194/jlr.R800049-JLR200. 18952570 PMC2674712

[8] Kim I, Xu W, Reed JC 2008 Cell death and endoplasmic reticulum stress: disease relevance and therapeutic opportunities. Nat Rev Drug Discov 7:1013–1030. doi:10.1038/nrd2755. 19043451

[9] Berridge MJ, Bootman MD, Roderick HL 2003 Calcium signalling: dynamics, homeostasis and remodelling. Nat Rev Mol Cell Biol 4:517–529. 12838335 10.1038/nrm1155

[10] Pinton P, Giorgi C, Siviero R, Zecchini E, Rizzuto R 2008 Calcium and apoptosis: ER-mitochondria Ca2+ transfer in the control of apoptosis. Oncogene 27:6407–6418. doi:10.1038/onc.2008.308. 18955969 PMC2844952

[11] Wu J, Kaufman RJ 2006 From acute ER stress to physiological roles of the unfolded protein response. Cell Death Differ 13:374–384. doi:10.1038/sj.cdd.4401840. 16397578

[12] Bravo-Sagua R, Rodriguez AE, Kuzmicic J, Gutierrez T, Lopez-Crisosto C, Quiroga C, Diaz-Elizondo J, Chiong M, Gillette TG, Rothermel BA, Lavandero S 2013 Cell death and survival through the endoplasmic reticulum-mitochondrial axis. Curr Mol Med 13:317–329. doi:10.2174/156652413804810781. 23228132 PMC4104517

[13] Bertolotti A, Zhang Y, Hendershot LM, Harding HP, Ron D 2000 Dynamic interaction of BiP and ER stress transducers in the unfolded-protein response. Nat Cell Biol 2:326–332. doi:10.1038/35014014. 10854322

[14] Gardner BM, Pincus D, Gotthardt K, Gallagher CM, Walter P 2013 Endoplasmic reticulum stress sensing in the unfolded protein response. Cold Spring Harb Perspect Biol 5:a013169. doi:10.1101/cshperspect.a013169. 23388626 PMC3578356

[15] Lai E, Teodoro T, Volchuk A 2007 Endoplasmic reticulum stress: signaling the unfolded protein response. Physiology 22:193–201. doi:10.1152/physiol.00050.2006. 17557940

[16] Teske BF, Wek SA, Bunpo P, Cundiff JK, McClintick JN, Anthony TG, Wek RC 2011 The eIF2 kinase PERK and the integrated stress response facilitate activation of ATF6 during endoplasmic reticulum stress. Mol Biol Cell 22:4390–4405. doi:10.1091/mbc.E11-06-0510. 21917591 PMC3216664

[17] DuRose JB, Tam AB, Niwa M 2006 Intrinsic capacities of molecular sensors of the unfolded protein response to sense alternate forms of endoplasmic reticulum stress. Mol Biol Cell 17:3095–3107. doi:10.1091/mbc.E06-01-0055. 16672378 PMC1483043

[18] Wang S, Kaufman RJ 2012 The impact of the unfolded protein response on human disease. J Cell Biol 197:857–867. doi:10.1083/jcb.201110131. 22733998 PMC3384412

[19] Harding HP, Zhang Y, Bertolotti A, Zeng H, Ron D 2000 Perk is essential for translational regulation and cell survival during the unfolded protein response. Mol Cell 5:897–904. doi:10.1016/S1097-2765(00)80330-5. 10882126

[20] Liu Z, Lv Y, Zhao N, Guan G, Wang J 2015 Protein kinase R-like ER kinase and its role in endoplasmic reticulum stress-decided cell fate. Cell Death Dis 6:e1822. doi:10.1038/cddis.2015.183. 26225772 PMC4650730

[21] Scheuner D, Vander Mierde D, Song B, Flamez D, Creemers JW, Tsukamoto K, Ribick M, Schuit FC, Kaufman RJ 2005 Control of mRNA translation preserves endoplasmic reticulum function in beta cells and maintains glucose homeostasis. Nat Med 11:757–764. doi:10.1038/nm1259. 15980866

[22] Ma K, Vattem KM, Wek RC 2002 Dimerization and release of molecular chaperone inhibition facilitate activation of eukaryotic initiation factor-2 kinase in response to endoplasmic reticulum stress. J Biol Chem 277:18728–18735. doi:10.1074/jbc.M200903200. 11907036

[23] Szegezdi E, Logue SE, Gorman AM, Samali A 2006 Mediators of endoplasmic reticulum stress-induced apoptosis. EMBO Rep 7:880–885. doi:10.1038/sj.embor.7400779. 16953201 PMC1559676

[24] Harding HP, Novoa I, Zhang Y, Zeng H, Wek R, Schapira M, Ron D 2000 Regulated translation initiation controls stress-induced gene expression in mammalian cells. Mol Cell 6:1099–1108. doi:10.1016/S1097-2765(00)00108-8. 11106749

[25] Ma Y, Brewer JW, Diehl JA, Hendershot LM 2002 Two distinct stress signaling pathways converge upon the CHOP promoter during the mammalian unfolded protein response. J Mol Biol 318:1351–1365. doi:10.1016/S0022-2836(02)00234-6. 12083523

[26] Zinszner H, Kuroda M, Wang X, Batchvarova N, Lightfoot RT, Remotti H, Stevens JL, Ron D 1998 CHOP is implicated in programmed cell death in response to impaired function of the endoplasmic reticulum. Genes Dev 12:982–995. doi:10.1101/gad.12.7.982. 9531536 PMC316680

[27] Oyadomari S, Koizumi A, Takeda K, Gotoh T, Akira S, Araki E, Mori M 2002 Targeted disruption of the Chop gene delays endoplasmic reticulum stress-mediated diabetes. J Clin Invest 109:525–532. doi:10.1172/JCI0214550. 11854325 PMC150879

[28] Song B, Scheuner D, Ron D, Pennathur S, Kaufman RJ 2008 Chop deletion reduces oxidative stress, improves beta cell function, and promotes cell survival in multiple mouse models of diabetes. J Clin Invest 118:3378–3389. doi:10.1172/JCI34587. 18776938 PMC2528909

[29] Malhotra JD, Miao H, Zhang K, Wolfson A, Pennathur S, Pipe SW, Kaufman RJ 2008 Antioxidants reduce endoplasmic reticulum stress and improve protein secretion. Proc Natl Acad Sci U S A 105:18525–18530. doi:10.1073/pnas.0809677105. 19011102 PMC2587584

[30] Thorp E, Li G, Seimon TA, Kuriakose G, Ron D, Tabas I 2009 Reduced apoptosis and plaque necrosis in advanced atherosclerotic lesions of Apoe−/− and Ldlr−/− mice lacking CHOP. Cell Metab 9:474–481. doi:10.1016/j.cmet.2009.03.003. 19416717 PMC2695925

[31] Tabas I, Ron D 2011 Integrating the mechanisms of apoptosis induced by endoplasmic reticulum stress. Nat Cell Biol 13:184–190. doi:10.1038/ncb0311-184. 21364565 PMC3107571

[32] Pennuto M, Tinelli E, Malaguti M, Del Carro U, D'Antonio M, Ron D, Quattrini A, Feltri ML, Wrabetz L 2008 Ablation of the UPR-mediator CHOP restores motor function and reduces demyelination in Charcot-Marie-Tooth 1B mice. Neuron 57:393–405. doi:10.1016/j.neuron.2007.12.021. 18255032 PMC2267889

[33] Brewer JW, Hendershot LM, Sherr CJ, Diehl JA 1999 Mammalian unfolded protein response inhibits cyclin D1 translation and cell-cycle progression. Proc Natl Acad Sci U S A 96:8505–8510. doi:10.1073/pnas.96.15.8505. 10411905 PMC17546

[34] Brewer JW, Diehl JA 2000 PERK mediates cell-cycle exit during the mammalian unfolded protein response. Proc Natl Acad Sci U S A 97:12625–12630. doi:10.1073/pnas.220247197. 11035797 PMC18814

[35] Hamanaka RB, Bennett BS, Cullinan SB, Diehl JA 2005 PERK and GCN2 contribute to eIF2alpha phosphorylation and cell cycle arrest after activation of the unfolded protein response pathway. Mol Biol Cell 16:5493–5501. doi:10.1091/mbc.E05-03-0268. 16176978 PMC1289396

[36] Nutt SL, Hodgkin PD, Tarlinton DM, Corcoran LM 2015 The generation of antibody-secreting plasma cells. Nat Rev Immunol 15:160–171. doi:10.1038/nri3795. 25698678

[37] Garen A, Kauvar L, Lepesant JA 1977 Roles of ecdysone in Drosophila development. Proc Natl Acad Sci U S A 74:5099–5103. doi:10.1073/pnas.74.11.5099. 16592466 PMC432107

[38] Sato T, Jigami Y, Suzuki T, Uemura H 1999 A human gene, hSGT1, can substitute for GCR2, which encodes a general regulatory factor of glycolytic gene expression in Saccharomyces cerevisiae. Mol Gen Genet 260:535–540. doi:10.1007/s004380050926. 9928932

[39] Gao Q, Srinivasan S, Boyer SN, Wazer DE, Band V 1999 The E6 oncoproteins of high-risk papillomaviruses bind to a novel putative GAP protein, E6TP1, and target it for degradation. Mol Cell Biol 19:733–744. doi:10.1128/MCB.19.1.733. 9858596 PMC83930

[40] Zhang Y, Chen J, Gurumurthy CB, Kim J, Bhat I, Gao Q, Dimri G, Lee SW, Band H, Band V 2006 The human orthologue of Drosophila ecdysoneless protein interacts with p53 and regulates its function. Cancer Res 66:7167–7175. doi:10.1158/0008-5472.CAN-06-0722. 16849563

[41] Kim JH, Gurumurthy CB, Naramura M, Zhang Y, Dudley AT, Doglio L, Band H, Band V 2009 Role of mammalian Ecdysoneless in cell cycle regulation. J Biol Chem 284:26402–26410. doi:10.1074/jbc.M109.030551. 19640839 PMC2785328

[42] Mir RA, Bele A, Mirza S, Srivastava S, Olou AA, Ammons SA, Kim JH, Gurumurthy CB, Qiu F, Band H, Band V 2015 A novel interaction of Ecdysoneless (ECD) protein with R2TP complex component RUVBL1 is required for the functional role of ECD in cell cycle progression. Mol Cell Biol 36:886–899. doi:10.1128/MCB.00594-15. 26711270 PMC4810467

[43] Pagliarini V, Giglio P, Bernardoni P, De Zio D, Fimia GM, Piacentini M, Corazzari M 2015 Downregulation of E2F1 during ER stress is required to induce apoptosis. J Cell Sci 128:1166–1179. doi:10.1242/jcs.164103. 25616897

[44] Xu SH, Huang JZ, Chen M, Zeng M, Zou FY, Chen D, Yan GR 2017 Amplification of ACK1 promotes gastric tumorigenesis via ECD-dependent p53 ubiquitination degradation. Oncotarget 8:12705–12716. doi:10.18632/oncotarget.6194. 26498357 PMC5355047

[45] Bourougaa K, Naski N, Boularan C, Mlynarczyk C, Candeias MM, Marullo S, Fahraeus R 2010 Endoplasmic reticulum stress induces G2 cell-cycle arrest via mRNA translation of the p53 isoform p53/47. Mol Cell 38:78–88. doi:10.1016/j.molcel.2010.01.041. 20385091

[46] Horejsi Z, Stach L, Flower TG, Joshi D, Flynn H, Skehel JM, O'Reilly NJ, Ogrodowicz RW, Smerdon SJ, Boulton SJ 2014 Phosphorylation-dependent PIH1D1 interactions define substrate specificity of the R2TP cochaperone complex. Cell Rep 7:19–26. doi:10.1016/j.celrep.2014.03.013. 24656813 PMC3989777

[47] Ahn S, Kim J, Hwang J 2013 CK2-mediated TEL2 phosphorylation augments nonsense-mediated mRNA decay (NMD) by increase of SMG1 stability. Biochim Biophys Acta 1829:1047–1055. doi:10.1016/j.bbagrm.2013.06.002. 23831331

[48] Boulon S, Marmier-Gourrier N, Pradet-Balade B, Wurth L, Verheggen C, Jady BE, Rothe B, Pescia C, Robert MC, Kiss T, Bardoni B, Krol A, Branlant C, Allmang C, Bertrand E, Charpentier B 2008 The Hsp90 chaperone controls the biogenesis of L7Ae RNPs through conserved machinery. J Cell Biol 180:579–595. doi:10.1083/jcb.200708110. 18268104 PMC2234240

[49] Boulon S, Pradet-Balade B, Verheggen C, Molle D, Boireau S, Georgieva M, Azzag K, Robert MC, Ahmad Y, Neel H, Lamond AI, Bertrand E 2010 HSP90 and its R2TP/Prefoldin-like cochaperone are involved in the cytoplasmic assembly of RNA polymerase II. Mol Cell 39:912–924. doi:10.1016/j.molcel.2010.08.023. 20864038 PMC4333224

[50] Horejsi Z, Takai H, Adelman CA, Collis SJ, Flynn H, Maslen S, Skehel JM, de Lange T, Boulton SJ 2010 CK2 phospho-dependent binding of R2TP complex to TEL2 is essential for mTOR and SMG1 stability. Mol Cell 39:839–850. doi:10.1016/j.molcel.2010.08.037. 20864032

[51] Kim SG, Hoffman GR, Poulogiannis G, Buel GR, Jang YJ, Lee KW, Kim BY, Erikson RL, Cantley LC, Choo AY, Blenis J 2013 Metabolic stress controls mTORC1 lysosomal localization and dimerization by regulating the TTT-RUVBL1/2 complex. Mol Cell 49:172–185. doi:10.1016/j.molcel.2012.10.003. 23142078 PMC3545014

[52] Zhao R, Kakihara Y, Gribun A, Huen J, Yang G, Khanna M, Costanzo M, Brost RL, Boone C, Hughes TR, Yip CM, Houry WA 2008 Molecular chaperone Hsp90 stabilizes Pih1/Nop17 to maintain R2TP complex activity that regulates snoRNA accumulation. J Cell Biol 180:563–578. doi:10.1083/jcb.200709061. 18268103 PMC2234237

[53] Yuan XS, Wang ZT, Hu YJ, Bao FC, Yuan P, Zhang C, Cao JL, Lv W, Hu J 2016 Downregulation of RUVBL1 inhibits proliferation of lung adenocarcinoma cells by G1/S phase cell cycle arrest via multiple mechanisms. Tumour Biol 37:16015–16027. doi:10.1007/s13277-016-5452-9. 27722820

[54] Suh HW, Yun S, Song H, Jung H, Park YJ, Kim TD, Yoon SR, Choi I 2013 TXNIP interacts with hEcd to increase p53 stability and activity. Biochem Biophys Res Commun 438:264–269. doi:10.1016/j.bbrc.2013.07.036. 23880345

[55] Lee S, Min Kim S, Dotimas J, Li L, Feener EP, Baldus S, Myers RB, Chutkow WA, Patwari P, Yoshioka J, Lee RT 2014 Thioredoxin-interacting protein regulates protein disulfide isomerases and endoplasmic reticulum stress. EMBO Mol Med 6:732–743. doi:10.15252/emmm.201302561. 24843047 PMC4203352

[56] Oslowski CM, Hara T, O'Sullivan-Murphy B, Kanekura K, Lu S, Hara M, Ishigaki S, Zhu LJ, Hayashi E, Hui ST, Greiner D, Kaufman RJ, Bortell R, Urano F 2012 Thioredoxin-interacting protein mediates ER stress-induced beta cell death through initiation of the inflammasome. Cell Metab 16:265–273. doi:10.1016/j.cmet.2012.07.005. 22883234 PMC3418541

[57] Oslowski CM, Urano F 2011 Measuring ER stress and the unfolded protein response using mammalian tissue culture system. Methods Enzymol 490:71–92. doi:10.1016/B978-0-12-385114-7.00004-0. 21266244 PMC3701721

[58] Harding HP, Zeng H, Zhang Y, Jungries R, Chung P, Plesken H, Sabatini DD, Ron D 2001 Diabetes mellitus and exocrine pancreatic dysfunction in perk−/− mice reveals a role for translational control in secretory cell survival. Mol Cell 7:1153–1163. doi:10.1016/S1097-2765(01)00264-7. 11430819

[59] Scheuner D, Song B, McEwen E, Liu C, Laybutt R, Gillespie P, Saunders T, Bonner-Weir S, Kaufman RJ 2001 Translational control is required for the unfolded protein response and in vivo glucose homeostasis. Mol Cell 7:1165–1176. doi:10.1016/S1097-2765(01)00265-9. 11430820

[60] Huang B, Bates M, Zhuang X 2009 Super-resolution fluorescence microscopy. Annu Rev Biochem 78:993–1016. doi:10.1146/annurev.biochem.77.061906.092014. 19489737 PMC2835776

[61] Gustafsson MG, Shao L, Carlton PM, Wang CJ, Golubovskaya IN, Cande WZ, Agard DA, Sedat JW 2008 Three-dimensional resolution doubling in wide-field fluorescence microscopy by structured illumination. Biophys J 94:4957–4970. doi:10.1529/biophysj.107.120345. 18326650 PMC2397368

[62] Hetz C, Chevet E, Oakes SA 2015 Proteostasis control by the unfolded protein response. Nat Cell Biol 17:829–838. doi:10.1038/ncb3184. 26123108 PMC5546321

[63] Bu Y, Diehl JA 2016 PERK integrates oncogenic signaling and cell survival during cancer development. J Cell Physiol 231:2088–2096. doi:10.1002/jcp.25336. 26864318 PMC4912452

[64] Ding X, Ma M, Teng J, Shao F, Wu E, Wang X 2016 Numb protects human renal tubular epithelial cells from bovine serum albumin-induced apoptosis through antagonizing CHOP/PERK pathway. J Cell Biochem 117:163–171. doi:10.1002/jcb.25261. 26096024

[65] Zhu X, Zelmer A, Kapfhammer JP, Wellmann S 2016 Cold-inducible RBM3 inhibits PERK phosphorylation through cooperation with NF90 to protect cells from endoplasmic reticulum stress. FASEB J 30:624–634. doi:10.1096/fj.15-274639. 26472337

[66] Sanson M, Auge N, Vindis C, Muller C, Bando Y, Thiers JC, Marachet MA, Zarkovic K, Sawa Y, Salvayre R, Negre-Salvayre A 2009 Oxidized low-density lipoproteins trigger endoplasmic reticulum stress in vascular cells: prevention by oxygen-regulated protein 150 expression. Circ Res 104:328–336. doi:10.1161/CIRCRESAHA.108.183749. 19106412

[67] Oommen D, Prise KM 2013 Down-regulation of PERK enhances resistance to ionizing radiation. Biochem Biophys Res Commun 441:31–35. doi:10.1016/j.bbrc.2013.09.129. 24103755

[68] Shiraishi H, Okamoto H, Yoshimura A, Yoshida H 2006 ER stress-induced apoptosis and caspase-12 activation occurs downstream of mitochondrial apoptosis involving Apaf-1. J Cell Sci 119:3958–3966. doi:10.1242/jcs.03160. 16954146

[69] Zhao X, Mirza S, Alshareeda A, Zhang Y, Gurumurthy CB, Bele A, Kim JH, Mohibi S, Goswami M, Lele SM, West W, Qiu F, Ellis IO, Rakha EA, Green AR, Band H, Band V 2012 Overexpression of a novel cell cycle regulator ecdysoneless in breast cancer: a marker of poor prognosis in HER2/neu-overexpressing breast cancer patients. Breast Cancer Res Treat 134:171–180. doi:10.1007/s10549-011-1946-8. 22270930 PMC3397230

[70] Dey P, Rachagani S, Chakraborty S, Singh PK, Zhao X, Gurumurthy CB, Anderson JM, Lele S, Hollingsworth MA, Band V, Batra SK 2012 Overexpression of ecdysoneless in pancreatic cancer and its role in oncogenesis by regulating glycolysis. Clin Cancer Res 18:6188–6198. doi:10.1158/1078-0432.CCR-12-1789. 22977192 PMC3551465

[71] Claudius AK, Romani P, Lamkemeyer T, Jindra M, Uhlirova M 2014 Unexpected role of the steroid-deficiency protein ecdysoneless in pre-mRNA splicing. PLoS Genet 10:e1004287. doi:10.1371/journal.pgen.1004287. 24722212 PMC3983036

[72] Rao RV, Peel A, Logvinova A, del Rio G, Hermel E, Yokota T, Goldsmith PC, Ellerby LM, Ellerby HM, Bredesen DE 2002 Coupling endoplasmic reticulum stress to the cell death program: role of the ER chaperone GRP78. FEBS Lett 514:122–128. doi:10.1016/S0014-5793(02)02289-5. 11943137 PMC3971841

[73] Visioli F, Wang Y, Alam GN, Ning Y, Rados PV, Nor JE, Polverini PJ 2014 Glucose-regulated protein 78 (Grp78) confers chemoresistance to tumor endothelial cells under acidic stress. PLoS One 9:e101053. doi:10.1371/journal.pone.0101053. 24964091 PMC4071032

[74] Fu Y, Li J, Lee AS 2007 GRP78/BiP inhibits endoplasmic reticulum BIK and protects human breast cancer cells against estrogen starvation-induced apoptosis. Cancer Res 67:3734–3740. doi:10.1158/0008-5472.CAN-06-4594. 17440086

[75] Wang M, Ye R, Barron E, Baumeister P, Mao C, Luo S, Fu Y, Luo B, Dubeau L, Hinton DR, Lee AS 2010 Essential role of the unfolded protein response regulator GRP78/BiP in protection from neuronal apoptosis. Cell Death Differ 17:488–498. doi:10.1038/cdd.2009.144. 19816510 PMC2822118

[76] Lee HK, Xiang C, Cazacu S, Finniss S, Kazimirsky G, Lemke N, Lehman NL, Rempel SA, Mikkelsen T, Brodie C 2008 GRP78 is overexpressed in glioblastomas and regulates glioma cell growth and apoptosis. Neuro Oncol 10:236–243. doi:10.1215/15228517-2008-006. 18403493 PMC2563046

[77] Chang YJ, Chen WY, Huang CY, Liu HH, Wei PL 2015 Glucose-regulated protein 78 (GRP78) regulates colon cancer metastasis through EMT biomarkers and the NRF-2/HO-1 pathway. Tumour Biol 36:1859–1869. doi:10.1007/s13277-014-2788-x. 25431258

[78] Xing X, Li Y, Liu H, Wang L, Sun L 2011 Glucose regulated protein 78 (GRP78) is overexpressed in colorectal carcinoma and regulates colorectal carcinoma cell growth and apoptosis. Acta Histochem 113:777–782. doi:10.1016/j.acthis.2010.11.006. 21156321

[79] Daneshmand S, Quek ML, Lin E, Lee C, Cote RJ, Hawes D, Cai J, Groshen S, Lieskovsky G, Skinner DG, Lee AS, Pinski J 2007 Glucose-regulated protein GRP78 is up-regulated in prostate cancer and correlates with recurrence and survival. Hum Pathol 38:1547–1552. doi:10.1016/j.humpath.2007.03.014. 17640713

[80] Xing X, Lai M, Wang Y, Xu E, Huang Q 2006 Overexpression of glucose-regulated protein 78 in colon cancer. Clin Chim Acta 364:308–315. doi:10.1016/j.cca.2005.07.016. 16182273

[81] Fernandez PM, Tabbara SO, Jacobs LK, Manning FC, Tsangaris TN, Schwartz AM, Kennedy KA, Patierno SR 2000 Overexpression of the glucose-regulated stress gene GRP78 in malignant but not benign human breast lesions. Breast Cancer Res Treat 59:15–26. doi:10.1023/A:1006332011207. 10752676

[82] Wang M, Kaufman RJ 2014 The impact of the endoplasmic reticulum protein-folding environment on cancer development. Nat Rev Cancer 14:581–597. doi:10.1038/nrc3800. 25145482

[83] Nagelkerke A, Bussink J, Mujcic H, Wouters BG, Lehmann S, Sweep FC, Span PN 2013 Hypoxia stimulates migration of breast cancer cells via the PERK/ATF4/LAMP3-arm of the unfolded protein response. Breast Cancer Res 15:R2. doi:10.1186/bcr3373. 23294542 PMC3672809

[84] Singh PK, Wen Y, Swanson BJ, Shanmugam K, Kazlauskas A, Cerny RL, Gendler SJ, Hollingsworth MA 2007 Platelet-derived growth factor receptor beta-mediated phosphorylation of MUC1 enhances invasiveness in pancreatic adenocarcinoma cells. Cancer Res 67:5201–5210. doi:10.1158/0008-5472.CAN-06-4647. 17545600

[85] Taha MS, Nouri K, Milroy LG, Moll JM, Herrmann C, Brunsveld L, Piekorz RP, Ahmadian MR 2014 Subcellular fractionation and localization studies reveal a direct interaction of the fragile X mental retardation protein (FMRP) with nucleolin. PLoS One 9:e91465. doi:10.1371/journal.pone.0091465. 24658146 PMC3962360

[86] Dallner G 1974 Isolation of rough and smooth microsomes—general. Methods Enzymol 31:191–201. doi:10.1016/0076-6879(74)31021-X. 4418022

[87] Mohibi S, Srivastava S, Wang-France J, Mirza S, Zhao X, Band H, Band V 2015 Alteration/deficiency in activation 3 (ADA3) protein, a cell cycle regulator, associates with the centromere through CENP-B and regulates chromosome segregation. J Biol Chem 290:28299–28310. doi:10.1074/jbc.M115.685511. 26429915 PMC4653685

[88] Engelsberg A, Hermosilla R, Karsten U, Schulein R, Dorken B, Rehm A 2003 The Golgi protein RCAS1 controls cell surface expression of tumor-associated O-linked glycan antigens. J Biol Chem 278:22998–23007. doi:10.1074/jbc.M301361200. 12672804

